# Refractory extra nodal follicular dendritic cell sarcoma in a young male exhibiting a marked response to gemcitabine and docetaxel: A case report and literature review

**DOI:** 10.3892/mi.2025.265

**Published:** 2025-08-26

**Authors:** Kashif Ali Sarwar, Muhammad Sohaib Nadeem

**Affiliations:** Department of Oncology, Combined Military Hospital/National University of Medical Sciences, Rawalpindi 46000, Pakistan

**Keywords:** docetaxel, gemcitabine, chemotherapy, follicular dendritic cell sarcoma, advanced-stage disease

## Abstract

Follicular dendritic cell sarcoma (FDCS) is a rare tumour derived from dendritic cells located in B-follicles that play a pivotal role in the adaptive immune response. Surgery is the mainstay of treatment for localized disease; however, the management of unresectable or advanced disease is less well-defined. To date, to the best of our knowledge, there is no established or preferred chemotherapeutic regimen, although a number of regimens (primarily used in lymphomas and sarcomas) have been utilized with suboptimal outcomes. The present study describes the case of a young male patient with advanced-stage unresectable extranodal FDCS that progressed while the patient was undergoing doxorubicin/ifosfamide chemotherapy. Given the limited treatment options available, the patient was subsequently advised to undergo chemotherapy with a combination of gemcitabine/docetaxel. He achieved a marked clinical, as well as radiological response, and an improvement in performance status was also observed. This observation, while having a potential therapeutic bearing, also supports the mesenchymal behaviour of this rare type of tumour. With a number of other recent reports of effective responses to gemcitabine/docetaxel, and an increasing amount of available literature demonstrating its mesenchymal origin, the tumour has been reclassified as ‘stroma-derived neoplasms of lymphoid tissues’. Consequently, the accumulation of such case reports can help establish a novel first-line therapeutic regimen for advanced-stage FDCS.

## Introduction

Follicular dendritic cell sarcoma (FDCS) is a rare malignant tumour that arises from the uncontrolled proliferation of dendritic cells in lymphoid and extralymphoidal tissues. It was first described in a series of 4 patients in 1986 as a novel tumour arising from dendritic cells in lymphoid follicles (1). Dendritic cells function as antigen-presenting cells and play crucial roles in initiating and regulating the human adaptive immune response by secreting molecules, such as CXCL13, thus helping recruit B- and T-lymphocytes, which then play their respective immune-related roles. The disease usually affects adults, with a median age at diagnosis of 47-50 years (2,3). This tumour can develop in nodal, as well as extranodal tissues all over the body, characterized by slow-growing, painless solid mass(es). The traditional literature has quoted that up to 60% of cases arise from lymph nodes. However, more recent reports demonstrate a predominantly extranodal occurrence, particularly in the liver and spleen (4). Colonic FDCS, although rare, is specifically more common among Asian populations, as per a previous study (5). Up to 20% of cases are sequelae of Castleman disease (6). Epstein-Barr virus is considered to play a critical role in the pathogenesis of inflammatory pseudotumour-like FDCS of the liver and spleen (5,7). Considering this variability in sites of origin, and a growing body of literature demonstrating mesenchymal behaviour, this tumour entity has been reclassified in the new category of ‘stroma-derived neoplasm of lymphoid tissue’ in the 5th Edition of the World Health Organization (WHO) classification of haematolymphoid neoplasms (8). Pathologically, low-grade types of this tumour are characterized by spindle or ovoid cells arranged in a fascicular, whorled, or storiform pattern infiltrated with small lymphocytes. In high-grade forms, cells may be arranged in diffuse and sheet-like patterns. Typical markers of dendritic cell differentiation are CD21, CD23 and CD35. CXCL13, clusterin, fascin and podoplanin are additional markers that are uniformly positive (4,9). A recent study demonstrated that the highest sensitivity and specificity was exhibited by CD21, CD35, CXCL3, follicular dendritic cell secreted protein (FDCSP) and serglycin (SRGN) (10). While radical resection is the standard therapy for patients with localized disease, the treatment of advanced or unresectable disease is less well-established. Owing to the traditional view of its lymphoid origin and recent evidence of mesenchymal behaviour, chemotherapies against both types of tissue have been used with limited success. An optimal regimen has yet to be established; however, the rarity of this disease is a hindrance. Therefore, the accumulation of case reports and case series is critical for establishing the preferred treatment regimen. The present study reports the treatment of young patient diagnosed with advanced-stage mesenteric FDCS treated with gemcitabine/docetaxel chemotherapy.

## Case report

The patient in the present study was a 48-year-old male, with no known comorbidities. He initially presented to the Combined Military Hospital, Rawalpindi, Pakistan in April, 2022, with a 2-month history of constipation and abdominopelvic pain. The pain was dull in character, moderate in intensity, non-shifting, non-radiating and aggravated by lifting weights. There were no associated B-symptoms. A physical examination revealed an ill-defined non-tender immobile mass in the right iliac fossa, reaching up to the right lumbar and umbilical regions. The patient provided no personal or family history of any genetic syndrome(s) or cancer(s). Baseline blood counts and complete metabolic profiles were within normal limits. Initial imaging, including an ultrasonography of the abdomen, and a contrast-enhanced computed tomography (CT) scan of thorax, abdomen and pelvis were carried out. Imaging revealed a well-defined, lobulated, heterogeneously enhancing solid cystic mass lesion measuring 17.5x10.8x14 cm in size. The mass was predominantly cantered in the right hemi-abdomen and pelvis, appeared to arise from the mesentery, and extended from upper border of the L4 vertebra superiorly to the S3 vertebra inferiorly. The lesion was displacing and abutting adjacent bowel loops. Although bilateral ureters and the bladder were displaced, they had clear fat planes from the mass lesion. No mesenteric lymphadenopathy, liver or spleen involvement, or distant metastases were observed. Image-guided biopsy was later performed which revealed a malignant neoplasia, that was initially diagnosed as dedifferentiated liposarcoma owing to diffuse MDM2 positivity on immunohistochemistry (IHC; data not shown; IHC was performed at the hospital laboratory). The tumour was composed of sheets of atypical oval-shaped clear cells having pleomorphic vesicular nuclei, clumped chromatin and few mitoses. Scattered atypical lipoblasts were also observed. The case of the patient was discussed in a multidisciplinary tumour board involving surgeons, oncologists, a histopathologist and a radiologist. He was scheduled for exploratory laparotomy as per the recommendations of the tumour board. Per-operatively, a huge abdominopelvic mass, moderate ascites and few pelvic peritoneal and gut deposits were found. Maximal debulking (including right hemicolectomy and ileostomy) was performed to relieve compression symptoms, and prevent future intestinal/ureteric obstruction. The operative notes of the surgeon mentioned a 15x15 cm bilobulated mass in the retroperitoneum with attachment to the mesentery and involving the caecum and ascending colon. Multiple satellite nodules/deposits were found throughout the pelvic peritoneum and small bowel loops. Samples were obtained from these deposits for a histopathological evaluation. The liver surface was tumour free at this point in time. On the basis of pre-operative scans and peri-operative findings, the final TNM stage was cT4cN0M1.

**Figure 1:**
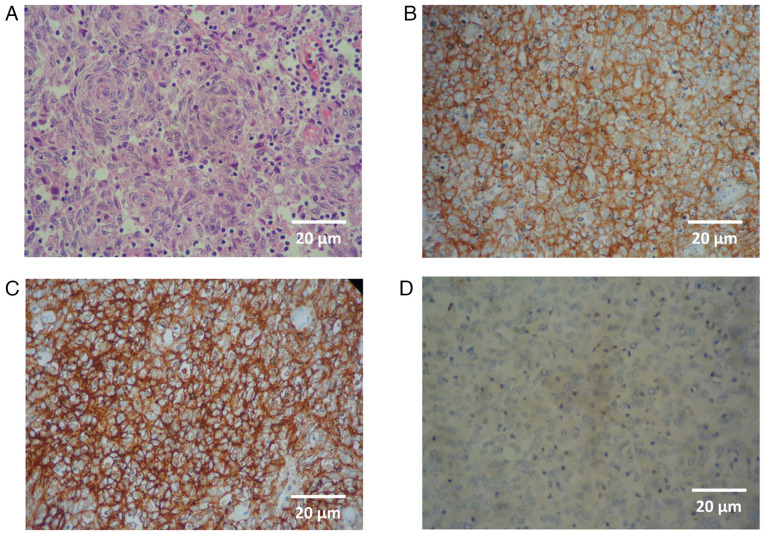
Histopathological analysis with immunohistochemistry (all images are shown at x400 magnification with corresponding scale bars at bottom right). (A) Haematoxylin and eosin staining illustrating a whorled pattern of dendritic cells with scattered lymphocytes. (B) Staining for CD21 illustrating strong reactivity. (C) Staining for CD23. (D) Weak staining for leukocyte common antigen (CD45).

The post-operative histopathological findings revealed dendritic cells arranged in a whorled pattern, with diffuse infiltration of lymphocytes on haematoxylin and eosin (H&E) staining. IHC revealed strong positivity for CD21 (reagent ref. no. PA0171, Leica Biosystems), CD23 (reagent ref. no PA0169, Leica Biosystems) and CD35 (reagent ref. no P-C021-70, Quartett Biotechnologie GmbH) and weak positivity for LCA (reagent ref. no PA0042, Leica Biosystems), as shown in Fig. 1. IHC yielded negative results for desmin, caldesmon, DOG1, S100, GATA3 and synaptophysin, hence supporting the diagnosis of FDCS (data not shown; IHC was performed at the hospital laboratory). Notably, both CD21 and CD23 were not applied on the initial biopsy. The final histopathology sample was also sent for reviewing to another academic cancer hospital laboratory (Shaukat Khanum Memorial Cancer Hospital and Research Centre-SKMC&RC) and the report obtained denoted FDCS. The regents and stains for both H&E and IHC were supplied by Leica Biosystems. For H&E staining, the ST Infinity H&E staining system partnered with the Leica ST 5010 stainer was used, while IHC was performed using the BOND RX Fully Automated Research Stainer. Steps were applied as detailed in the leicabiosystems.com knowledge pathway.

**Figure 2:**
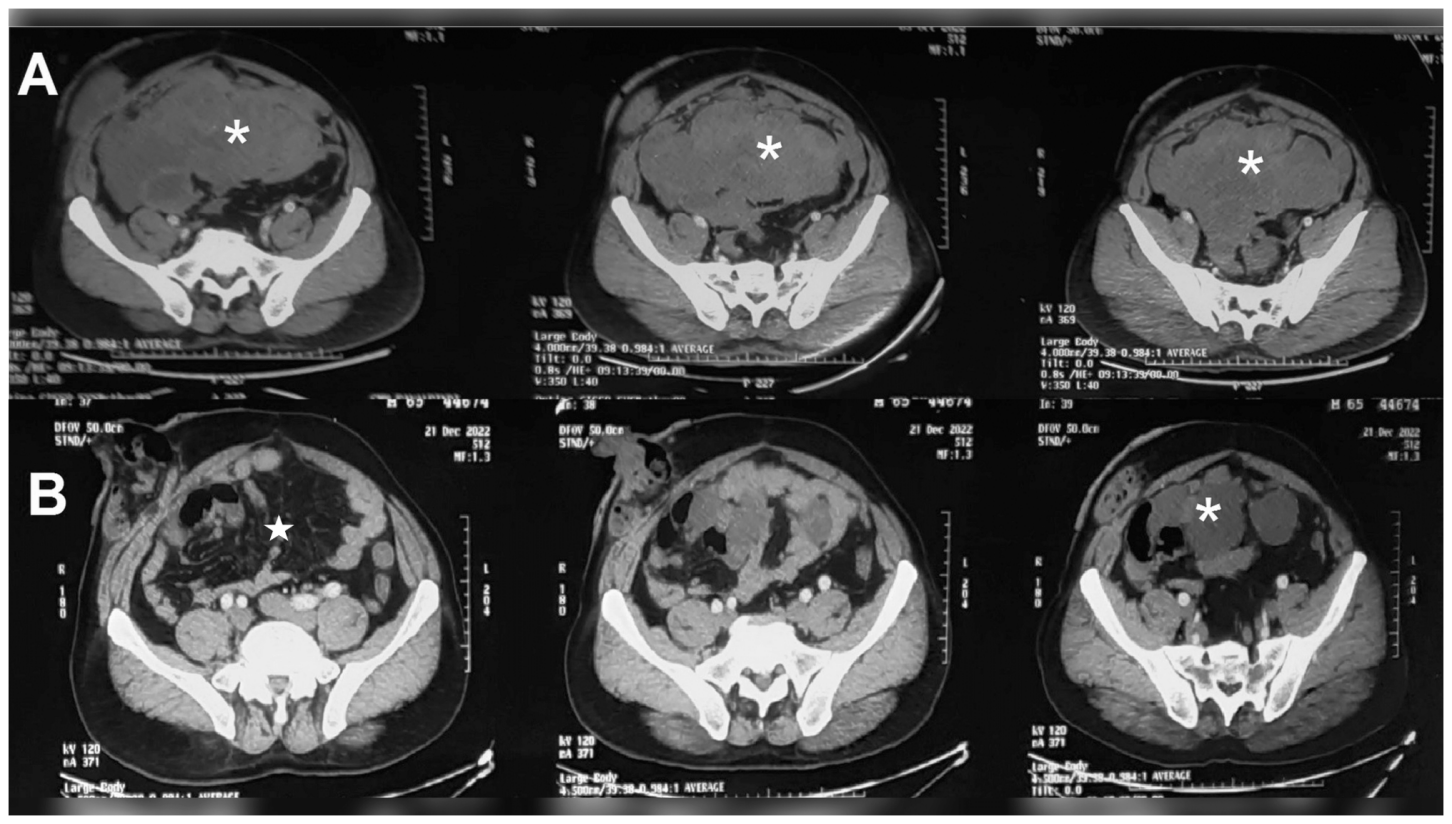
Representative slices of contrast-enhanced computed tomography illustrating tumour [asterisk symbol (*)] (A) prior to gemcitabine/docetaxel chemotherapy and (B) following 3 months of chemotherapy. (A) A well-defined, lobulated, heterogeneously enhancing solid cystic mass lesion can be observed, measuring 19.2x16.6x14.3 cm (obtained on October 3, 2022). (B) A marked reduction in the size of same mass to 14x12.4x10 can be observed (obtained on December 21, 2022). The mass lesion illustrated by an asterisk (*) is not visible in first image of the 2nd row (LV5-SV1 level). The clear mesentery can be seen marked by a white triangle. In the other two images, only small cystic masses can be seen indicating significant reduction of overall tumour size at these levels (Lower SV1 and SV2, respectively).

The samples from the peritoneal and gut deposits exhibited metastasis. The tumour also contained some necrotic (40%) and haemorrhagic areas, which bears prognostic value. Following recovery and adequate wound healing, the patient was offered 3 weekly cycles of doxorubicin (20 mg/m^2^) and ifosfamide/mesna (both 3 gm/m^2^) on days 1,2 and 3 (The Clatterbridge Cancer Centre NHS Trust protocol ref: MPHADOXIFO). Five 3-weekly courses were administered, and the response evaluation CT scan revealed progressive disease in terms of the size of both primary and peritoneal deposits, as well as the appearance of multiple subcapsular liver lesions in segment IVa and VI. The maximum size of the primary lesion was 19.2x16.6x14.3 cm now (scan dated October 3, 2022 as shown in the top row of Fig. 2), along with the development of moderate ascites. Chemotherapy was terminated owing to the progression of the disease, and achieving a lifetime cumulative dose of doxorubicin. Following a 5-week break from chemotherapy, the case of the patient was re-discussed in the intradepartmental tumour board, and was placed on second-line gemcitabine and docetaxel. Gemcitabine at 1000 mg/m^2^ alone was administered on day 1, and in combination with docetaxel (at 75 mg/m^2^) on day 8; both were administered in 3-weekly chemotherapy cycles. During chemotherapy, the patient began to experience improvement in pain, as well as in abdominal distension. Following the completion of the fourth cycle of chemotherapy, he was advised to undergo a response evaluation scan and the results revealed a marked objective decrease in the size of the lesion, as well as the resolution of ascites, as illustrated in the representative CT scan images presented in Fig. 2. The maximum tumour size decreased to 14x12.4x10 cm following only 12 weeks of chemotherapy, from a size of 19.2 x16.6x14.3 cm. His performance status markedly improved, and no grade ≥3 toxicities appeared. Only grade 2 hand-foot syndrome and grade 1 neutropenia occurred, which were treated conservatively.

Following the completion of 24 weeks (six 3-weekly cycles) of gemcitabine/docetaxel chemotherapy, the tumour further regressed, with the maximum size decreasing to 9x6x6.1 cm on the response evaluation CT scan performed 4 weeks following the completion of chemotherapy (images not available). The liver metastases were stable, suggesting a continued response. The patient became asymptomatic and was routinely performing his home and occupational activities of daily living. Following discussions with the multi-disciplinary team and with the patient, he was maintained on surveillance with serial contrast-enhanced CT scan evaluations every 12 weeks. The updated RECIST v1.1 stable disease status remained until 6 months following the completion of gemcitabine/docetaxel chemotherapy, after which his disease began to progress. The patient was then commenced on cyclophosphamide/vincristine/doxorubicin/prednisone (CHOP) regimen; however, the disease progressed clinically after two cycles. Considering the limited treatment options, treatment with oral pazopanib 400 mg once daily was selected. However, he tolerated this treatment poorly and it was terminated after 4 weeks. He required repeated red cell transfusions owing to bone marrow suppression. Moreover, his general condition and performance status deteriorated as the disease progressed. The patient was placed on palliative care; regrettably, he eventually succumbed due to disease progression and bone marrow failure in June, 2024, almost 2 years following his diagnosis.

The present study was conducted according to the principles of the Declaration of Helsinki and informed consent for publication was obtained from the patient while he was alive.

## Discussion

FDCS is a rare tumour. Its origin, diagnosis and management has been an enigma since it was first described. It is often misdiagnosed on an initial biopsy, adding to the complexity of managing this disease (11). Traditional chemotherapies are mostly ineffective, and/or carry significant toxicity with no standard regimen for advanced disease to date. In a previous study, extranodal FDCS accounted for 37% of all new cases of FDCS. The same study also reported that extranodal origin, bulky disease and intra-abdominal disease were associated with inferior PFS and OS (12). Another study cited the following poor prognostic factors: Size of the tumour (> 6 cm), nuclear pleomorphism, increased mitoses (>5/10 high power fields), necrosis and intra-abdominal location (13). The same information has also been incorporated in the WHO 5th edition. The patient in the present study had almost all of the aforementioned characteristics associated with adverse survival outcomes. He was offered gemcitabine/docetaxel combination and the results revealed marked radiological regression of the tumour and a moderately sustained clinical response.

As of 2021, a total of 69 cases of gastrointestinal FDCS have been reported in the English literature (3), although the total number of reported cases is a few hundred in all body sites. Since it was first described in lymph node follicles (1), and hence obtained its name, it has been considered to have a lymphoid origin due to its cited predominance in lymph nodes in conventional literature, and some association with Castleman disease and autoimmune diseases (12). FDCS has also been associated with lymphoid and myeloid neoplasia, and evidence of transdifferentiation has been noted (14). On the basis of this understanding of the origin of this tumour, regimens designed to treat high grade lymphomas were used with variable success rates. These include CHOP, ifosfamide/carboplatin/etoposide (ICE), and doxorubicin/bleomycin/vincristine/dacarbazine (ABVD) (3,15,16). Even bendamustine has been tried in a previous report of a case of pancreatic FDCS that progressed after multiple lines of chemotherapy (17). As aforementioned, the mesenchymal origin of FDCS has also been hypothesized due to its mesenchymal behaviour (4,16). Some researchers also describe follicular dendritic cell precursors to be of ubiquitous perivascular stromal origin, rather than of lymphoid origin (18). Over the years, evidence of a stromal rather than a lymphoid origin has come to light. In addition to well-established markers such as CD21, CD23 and CD35, novel IHC markers, such as somatostatin receptor 2, FDCSP and SRGN have gained recognition to aid in the diagnosis and understanding of origin (10). All these recent developments have led to the reclassification of FDCS. In the revised 4th edition of the WHO classification, FDCS was classified under ‘histiocytic/dendritic cell neoplasms’, but has now been reclassified in WHO 5th edition under ‘mesenchymal dendritic cell neoplasms’ in the new category of ‘stroma-derived neoplasms of lymphoid origin’ alongside other similar entities e.g., intranodal palisaded myofibroblastoma (8). With this in view, the doxorubicin/ifosfamide combination, and doxorubicin alone have also been utilized due to their success in treating sarcomas in general. However, all the chemotherapies provide short-term relief, if at all, with significant treatment-related toxicities and high rates of failure. The gemcitabine/docetaxel regimen has traditionally been employed in second-line setting for various soft tissue sarcomas and has relatively good tolerability (19). The maximum benefit was achieved in leiomyosarcomas, with response rates reaching 50%, although poor responses were observed in other subtypes. Considering the mesenchymal behaviour of this rare tumour type, it was logical to use this regimen in FDCS following suboptimal responses from the doxorubicin/ifosfamide combination or doxorubicin alone. The first report on the objective response of FDCS to gemcitabine/docetaxel combination was described in 2 patients by Conry (20). One patient had mesenteric FDCS, and the other had duodenal wall FDCS. The author also reported good tolerance. This was followed by two similar reports of good responses in two patients who had disease in the liver and neck, and were treated with the same regimen (21,22). The case described in the present study further supports its use in advanced-stage or unresectable FDCS of the GI tract and related organs, and reinforces the mesenchymal behaviour of this rare tumour.

It has also been shown that a greater proportion of patients with FDCS express PD-L1 and PD-L2(23). Previously, Vermi *et al* reported a prominent role of EGFR signalling in the development and proliferation of FDCs and indicated that blockade of this receptor might be clinically relevant and explored (24). This may open up avenues for treatment with immune checkpoint inhibitors and/or tyrosine-kinase inhibitors in the future, although no data are available to date that have evaluated their potential role in the treatment of either localized or advanced stages of this rare disease.

In conclusion, to the best of our knowledge, the present case report is among the very few involving patients with advanced-stage FDCS was treated with gemcitabine/docetaxel. Overall, the patients achieved excellent clinical and radiological responses, as well as good tolerance. Since FDCS is very rare and randomized controlled trials may be impractical to conduct, the further accumulation of such cases/series is warranted, as these pooled data can potentially be practice changing, and may lead to a recommendation of gemcitabine/docetaxel as a first-line option in advanced-stage FDCS.

## Data Availability

The data generated in the present study may be requested from the corresponding author.

## Authors' information

The ORCID IDs of the authors are: KAS (https://orcid.org/0000-0001-7746-0724) and MSN (https://orcid.org/0000-0002-8546-1994).
